# Trends and Outcomes of Surgical Treatment for Colorectal Cancer between 2004 and 2012- an Analysis using National Inpatient Database

**DOI:** 10.1038/s41598-017-02224-y

**Published:** 2017-05-17

**Authors:** Meng-Tse Gabriel Lee, Chong-Chi Chiu, Chia-Chun Wang, Chia-Na Chang, Shih-Hao Lee, Matthew Lee, Tzu-Chun Hsu, Chien-Chang Lee

**Affiliations:** 1 Department of Emergency Medicine, National Taiwan University Hospital, Taipei, Taiwan; 2 Department of General Surgery, Chi Mei Medical Center, Tainan and Liouying, Taiwan; 3 Department of Electrical Engineering, Southern Taiwan University of Science and Technology, Tainan, Taiwan; 4 Division of Radiation Oncology, Department of Oncology, National Taiwan University Hospital, Taipei, Taiwan; 5 Graduate Institute of Epidemiology and Preventive Medicine, College of Public Health, National Taiwan University, Taipei, Taiwan; 6 Department of Radiation Oncology, Taipei Municipal Wan-Fang Hospital, Taipei, Taiwan; 7 Medical Wisdom, Taxas, USA

## Abstract

Limited data are available for the epidemiology and outcome of colorectal cancer in relation to the three main surgical treatment modalities (open, laparoscopic and robotic). Using the US National Inpatient Sample database from 2004 to 2012, we identified 1,265,684 hospitalized colorectal cancer patients. Over the 9 year period, there was a 13.5% decrease in the number of hospital admissions and a 43.5% decrease in in-hospital mortality. Comparing the trend of surgical modalities, there was a 35.4% decrease in open surgeries, a 3.5 fold increase in laparoscopic surgeries, and a 41.3 fold increase in robotic surgeries. Nonetheless, in 2012, open surgery still remained the preferred surgical treatment modality (65.4%), followed by laparoscopic (31.2%) and robotic surgeries (3.4%). Laparoscopic and robotic surgeries were associated with lower in-hospital mortality, fewer complications, and shorter length of stays, which might be explained by the elective nature of surgery and earlier tumor grades. After excluding patients with advanced tumor grades, laparoscopic surgery was still associated with better outcomes and lower costs than open surgery. On the contrary, robotic surgery was associated with the highest costs, without substantial outcome benefits over laparoscopic surgery. More studies are required to clarify the cost-effectiveness of robotic surgery.

## Introduction

Colorectal cancer is the fourth most commonly diagnosed cancer in the United States in 2013. According to the 2010–2012 National Cancer Institute cancer fact sheet, approximately 4.5% of the US population will be diagnosed with colorectal cancer at some point during their lifetime^1^. Notably from 2003 to 2012, there has been an approximately 30% decline in both the incidence and mortality of this common disease^1, 2^. This dramatic decline in the incidence of colorectal cancer has been largely attributed to the increase adoption of fecal-occult-blood test (FOBT) and colonoscopy screening, which allows early detection and removal of adenomatous polyps^3–5^. The decrease in colorectal cancer mortality is likely due to a combined effects of several factors such as increase in adoption of colorectal cancer screening^6, 7^, changes in risk factors for colorectal cancer^5, 8, 9^, and improvement in colorectal cancer treatment^5, 10–12^.

Surgery is the most common treatment for resectable colorectal cancer, and during the last decade, it has experienced some major improvements on pre-operative assessment, instrument, surgical techniques, intra-operative monitor and post-operative care. Traditionally, colorectal cancer was removed through large open abdominal incisions. In recent years, there has been a widespread shift toward the minimally invasive surgery (MIS)^13–17^. Numerous meta-analyses have shown that laparoscopy surgery offers numerous advantages to its open counterpart, for example, fewer wound complications, quicker return of bowel function and normal diet, shorter postoperative hospitalization, and faster recovery^18–20^. Recently, the introduction of robot-assisted surgery improves on the limitation of laparoscopy surgery by allowing better vision, precision and, dexterity of movement^21–23^. However, there are still limited nation-wide studies on the comparisons between laparoscopic and robotic surgeries, as specific ICD9-CM codes for these procedures were only introduced in 2008.

As far as we were aware of, most of the epidemiology data on colorectal cancer came from the National Cancer Institute’s Surveillance, Epidemiology, and End Results (SEER) program registries. SEER Program registries collect data from 18 geographic areas across the United States and is not a true nation-wide data. Thus, we are interested in giving a comprehensive epidemiology overview of colorectal cancer admission in the United States from 2004 through 2012.

## Methods

### Data Sources

This study is conducted using 2002–2012 data from the Nationwide Inpatient Sample (NIS), which is compiled and distributed by the Healthcare Cost and Utilization Project (HCUP). The NIS is the largest all-payer inpatient database in the US, which estimates more than 35 million hospitalizations by sampling approximately 20% of all US hospital discharges^24^. Before 2012, the NIS comprised of all inpatient discharges (100%) by randomly sampling 20% of hospitals. NIS was updated in 2012 to systematically sampled 20% of discharges from all hospitals (100%). We used the new set of weights called “trend weights” that were developed by HCUP for the 2012 data, as well as for data for previous years (1993–2011)^25^.

Since the NIS database contains de-identified information regarding each hospitalization, the need for informed consent was waived. This study was approved by the institutional review board of Chi Mei Medical Center.

### Definitions and Variables

Clinical Classifications Software (CCS) is a tool developed by Agency for Healthcare Research and Quality (AHRQ) for clustering patient diagnoses and procedures into a manageable number of clinically meaningful categories. Patients were defined to have colorectal cancer if they had a primary diagnosis of either CCS code 14 or 15. Since CCS code 15 contains ICD9-CM codes for anal cancer, we excluded the anal cancer codes in our analysis. Patients were identified to have a colorectal surgical procedure by the above diagnosis codes plus any colorectal procedures codes for open, laparoscopic and robotic surgery. The specific (ICD-9CM) procedure codes used for identification of open colorectal cancer procedure were 45.71–79, 45.82–83, 48.40–49, 48.50–59, and 48.61–69, used for identification of laparoscopic procedures were 17.31–39, 45.81, 48.42, and 48.51 and used for identification of robotic procedures were 17.41–49.

Patient characteristics, information about their hospital stay, information on elective VS emergent admission, pathologic staging, and in-hospital outcomes were used as coded in the NIS, unless stated otherwise. We followed the previously reported classification of pathologic staging: localized disease (AJCC Stage 1, NIS 1.01, 2.01), locally advanced disease or symptoms (AJCC Stage 2, NIS 2.02, 2.03, 2.04), regional nodal disease (AJCC Stage 3, NIS 3.01), or metastatic disease (AJCC Stage IV, NIS 3.02)^26^.

For Elixhauser comorbidity scores calculation, we used the approach developed by Thompson *et al*., which consisted of 29 comorbidities^27^. The outcomes investigated in this study were in-hospital mortality, the length of hospital stay, in-hospital complications, and overall cost of hospitalization. We compared the outcomes before and after excluding patients with the locally advanced disease or symptoms, regional nodal disease, metastatic disease, and emergent pathology. Previously reported exclusion criteria are used^26^. Briefly, emergent admissions, pathology associated with volvulus fistula formation, gross perforation, peritonitis, and shock are excluded.

Three types of hospital complications were identified by ICD-9-CM Codes: wound complications (998.3, 879.2, 998.1), general medical complications (410, 411.81, 427.9, 518.0, 518.81), and general surgical complications (560.1, 567, 568.81, 998.5). The cost was estimated by multiplying hospital charges with the cost-to-charge ratios for a given year. Both the hospital charges and the cost-to-charge ratio are provided by HCUP.

### Statistical Analysis

Survey data were analyzed using the recommendations from AHRQ. Descriptive statistics were obtained using survey-specific statements such as SURVEYMEANS. Patient-specific and hospital-specific discharge weights were used to obtain national estimates. Characteristics and outcomes for the study population were reported using percentages for categorical variables. For continuous variables, they were reported using medians together with interquartile ranges or mean with standard error. Survey-weighted Cox proportional hazard regression models were used to assess the association between the type of surgery and in-hospital mortality, adjusting for patient demographics, income, comorbidity score, disease stage, hospital characteristics, and type of admission^28^. All analyses and plots were conducted using SAS 9.3 for Windows (SAS Institute Inc, Cary, NC, USA).

### Ethical approval

This study is approved by institutional review board of Chi Mei Medical Center.

## Results

### Incidence and baseline characteristics

**Figure 1:**
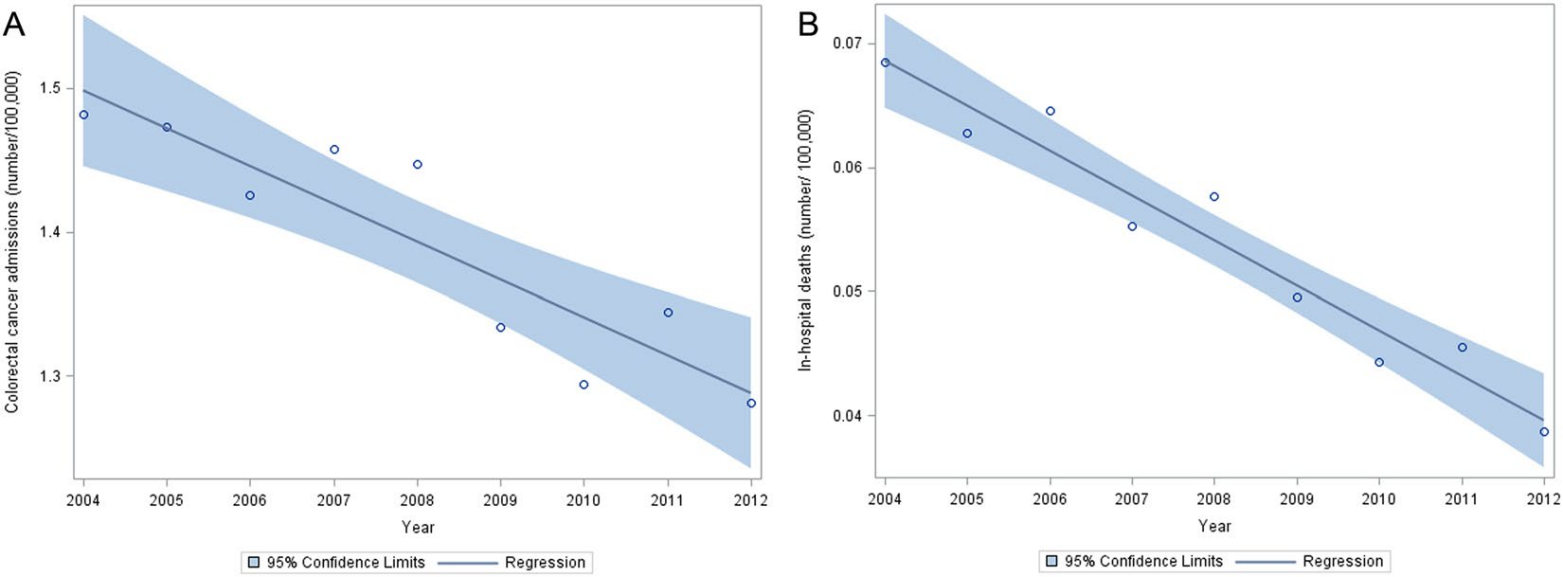
The trend of colon cancer admission (**A**) and in-hospital mortality (**B**) between 2004 and 2012. Circle represents the number of admissions/deaths in a given year, the straight line is a linear regression on all the data points, and the shaded area represents the 95% confidence limits for the linear regression.

From 2004 to 2012, we identified 1,265,684 weighted number of colorectal cancer admissions. Figure 1 shows that during the study period, there are a 13.5% decrease in the number of colorectal cancer patients (from 1.48 per 100,000 admissions in 2004 to 1.28 per 100,000 admissions in 2012), and 43.5% decrease in the number of deaths (from 0.069 deaths per 100,000 colorectal cancer admissions to 0.039 deaths per 100,000 colorectal cancer admissions in 2012). The baseline characteristics of these patients are shown in Table 1. Most of the patients that were admitted were elderly (>65 years old), and were at a localized stage of cancer (57.8%).

**Table 1:** Characteristics of the study cohort over three time periods.

Characteristic	2004–2006	2007–2009	2010–2012
Annual number of admissions	438015	423787	403882
Demographics			
Male sex, no (%)	218208 (49.9%)	214746 (50.76%)	204465 (50.67%)
Age (Mean ± SE)	68.65 ± 0.105	67.90 ± 0.137	67.29 ± 0.118
Income			
Income quartile 1, no (%)	109542 (25.52%)	107084 (25.83%)	107918 (27.27%)
Income quartile 2, no (%)	111779 (26.04%)	109658 (26.45%)	99466 (25.14%)
Income quartile 3, no (%)	105967 (24.69%)	98883 (23.85%)	98443 (24.88%)
Income quartile 4, no (%)	101811 (23.72%)	98815 (23.84%)	89781 (22.69%)
Comorbidity			
Combined comorbidity score (Mean ± SE)	3.01 ± 0.039	3.23 ± 0.055	3.68 ± 0.05
Disease stage			
Localized	245456 (56.03%)	244713 (57.74%)	241488 (59.79%)
Locally advanced	22013 (5.02%)	21701 (5.12%)	22052 (5.46%)
Regional nodal disease	64827 (14.80%)	54081 (12.76%)	40749 (10.08%)
Metastatic	100029 (22.83%)	96983 (22.88%)	92864 (22.99%)
Outcome			
Mortality, no (%)	19588 (4.47%)	16244 (3.84%)	13385 (3.31%)
Length of stay (Mean ± SE)	8.78 ± 0.049	8.42 ± 0.043	7.952 ± 0.039
Total charge for inhospital stay (Mean ± SE)	44881 ± 695.83	56238 ± 898.67	66546 ± 925.70
Hospital characteristics			
Rural hospital, no (%)	61460 (14.04%)	51300 (12.17%)	45763 (11.41%)
Urban, nonteaching hospital, no (%)	194276 (44.38%)	177782 (42.19%)	156853 (39.14%)
Urban, teaching hospital, no (%)	181939 (41.56%)	192208 (45.62%)	198170 (49.44%)

During the study period, there is little trend change in age, sex, and income of colorectal cancer patients. However, we observed, 46.4% decrease in the number of regional nodal cases, 27.5% increase in the cost of hospitalization, 11.2% decrease in the mean length of stay, and 37.7% increase in the mean combined comorbidity score from the year 2002 to the year 2012. The combined comorbidity score consists of 29 comorbidities from the Elixhauser comorbidity system (acquired immunodeficiency syndrome, alcohol abuse, deficiency anemia, rheumatoid arthritis, chronic blood loss anemia, congestive heart failure, chronic pulmonary disease, coagulopathy, depression, diabetes without complications, diabetes with chronic complications, drug abuse, hypertension (uncomplicated and complicated), hypothyroidism, liver disease, lymphoma, fluid and electrolyte disorders, metastatic cancer, neurological disorders, obesity, paralysis, peripheral vascular disorders, psychoses, pulmonary circulation disorders, renal failure, solid tumor without metastasis, peptic ulcer disease, valvular disease, and weight loss), and has been found to predict in-hospital mortality with high frequency. In addition, we found an increase in the number of colorectal cancer admissions to urban teaching hospitals, and a corresponding decrease in the number of admissions to urban non-teaching and rural hospitals.

### Trends in the volume of the different types of surgical procedures

**Figure 2:**
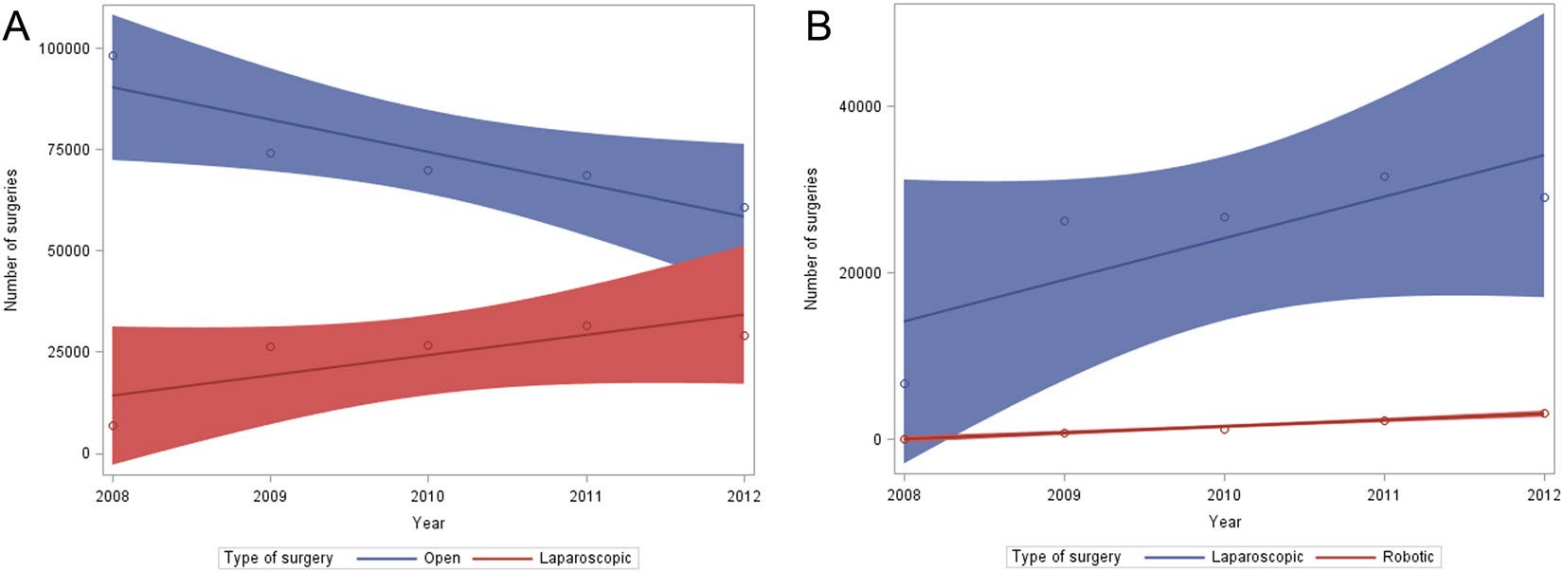
The number of colorectal surgeries, according to the type of surgeries, from 2008 to 2012. (**A**) Trends in the number of open vs. laparoscopic surgery (**B**) Trend in the number of laparoscopic vs robotic surgery. Circle represents the number of surgeries in a given year, the straight line is a linear regression on all the data points, and the shaded area represents the 95% confidence limits for the linear regression.

Figure 2 compares the trend of different surgical treatment modalities for colorectal cancer inpatients ((A.) open vs. laparoscopic surgery, (B) laparoscopic vs robotic surgery). From 2004 to 2012, there was a 40.5% decrease in the number of open colorectal procedure (from 1.02 per 100,000 procedures in 2004 to 0.61 per 100,000 procedures in 2012). Specific ICD9-CM codes for laparoscopic and robotic surgery were introduced in the year 2008, so the comparisons of these procedures were made from the year 2008. We observed that from 2008 to 2012, there was 35.4% decrease in the number of open surgeries (94,190 to 60,855), 3.5-fold increase in the number of colorectal laparoscopic surgeries (6536 to 29,105) and 41.3 fold increase in the number of colorectal robotic surgeries (74 to 3130). In 2012, open surgery is still the most common surgical treatment (65.4%), followed by laparoscopic (31.2%) and finally robotic surgeries (3.4%).

### Patient characteristics and outcome of different surgical procedures

Table 2 aggregates patient characteristics and outcomes from the same surgical treatment modality from 2008 to 2012. The three types of surgeries were mainly performed on elective admission (open 95.8%, laparoscopic 98.4%, and 99.6%) and patients with localized cancer (open 61.4%, laparoscopic 74.3% and robotic 78.4%). However, open surgery was selected against the other surgeries for regional or distant cancer, and emergency surgery. Open surgeries have the highest percentage of patients with emergent pathology, locally advanced disease, regional nodal disease, and metastatic disease. For the type of hospital conducting the surgery, most of the surgeries are conducted in urban teaching hospital and few surgeries are conducted in rural hospital. When the different types of surgeries that are conducted in rural hospital are compared, we observed that open surgery (46806/371463 = 12.69%) has the highest number, followed by laparoscopic surgery (9135/120615 = 7.63%), and finally robotic surgery (83/7646 = 1.08%).

**Table 2:** Characteristics and outcomes of 3 different types of colorectal surgeries from 2008–2012.

Characteristics	Open N = 371463	Laparoscopic N = 120615	Robotic N = 7646
Type of admission			
Elective admission	355870 (95.8%)	118790 (98.4%)	7620 (99.6%)
Emergent admissions	15593 (4.19%)	1825 (1.51%)	26 (0.34%)
Pathology			
Emergent	12517 (3.36%)	1677 (1.39%)	72 (0.94%)
Localized	228223 (61.43%)	89609 (74.29%)	5995 (78.4%)
Locally advanced	15391 (4.14%)	4554 (3.77%)	195 (2.55%)
Regional nodal disease	53782 (14.47%)	15049 (12.47%)	736 (9.62%)
Metastatic	61507 (16.55%)	9726 (8.06%)	648 (8.47%)
Outcome			
Mortality, no (%)	8861 (2.387%)	1143 (0.948%)	19 (0.257%)
Length of stay, (Mean ± SE), Days	9.30 ± 0.04	6.47 ± 0.046	6.32 ± 0.14
No of complications. (%)	125574 (33.79%)	30473 (25.25%)	1676 (21.9%)
Total cost, median (IQR), USD	$16,486 (11572, 24973)	$13,844 (10224, 19777)	$19,185 (14251, 25990)
Cost per day, median (IQR), USD	$2,217.93 (1737.66, 2906.05)	$2,669.64 (2062.87, 3535.61)	$3,749.81 (2764.84, 5061.40)
Hospital characteristics			
Rural hospital, no (%)	46806 (12.69%)	9134.98 (7.63%)	82.919 (1.08%)
Urban, nonteaching hospital, no (%)	150433 (40.8%)	50605 (42.31%)	2309.36 (30.33%)
Urban, teaching hospital, no (%)	171463 (46.5%)	59850 (50.04%)	5219 (68.57%)

In general, colorectal cancer patients undergoing open surgery has the worst in-hospital outcome. When compared to either laparoscopic or robotic surgery, open surgery has higher length of hospital stay, higher in-hospital mortality rate and more number of total complications (wound complications, general medical complications, and general surgical complications). However, the outcomes of laparoscopic and robotic surgery are comparable. In terms of overall total cost, robotic surgery was the most expensive (median = $19,185), followed by open surgery (median = $16,486) and finally laparoscopic surgery (median = $13,844).

### Outcomes of different surgical procedures in patients with localized disease

Patients with emergent admission and advanced stage of colorectal have a poor prognosis, and we wanted to investigate if excluding these patients will change the outcome comparison of the different surgical procedures. Table 3 aggregates all elective surgeries for localized pathology from 2008 to 2012, and stratifies by the three types of surgeries. Patients undergoing open surgery were still associated with the worst outcome. When compared to either laparoscopic or robotic surgery, open surgery has a higher length of hospital stay, higher in-hospital mortality rate, and a higher number of complications. However, the outcomes of laparoscopic and robotic surgery are comparable. In terms of overall total cost, robotic surgery was still the most expensive (median = $18,940), followed by open surgery (median = $14,807) and finally laparoscopic surgery (median = $13,023).

**Table 3:** Comparison of elective surgeries for localized pathology.

Outcome	Open N = 222226	Laparoscopic N = 88707	Robotic N = 5970
Mortality, No. (%)	2756 (1.24%)	461 (0.52%)	14 (0.24%)
Length of hospital stay, (Mean ± SE), Days	8.06 ± 0.03	5.82 ± 0.04	5.97 ± 0.14
No of complications. (%)	63442 (28.54%)	18761 (21.14%)	1219 (20.42%)
Total cost, median (IQR), USD	14807 (10783, 21612)	13023 (9847, 18103)	18940 (13982, 25280)
Cost per day, median (IQR), USD	2234 (1746, 2920)	2702 (2084, 3583)	3886 (2842, 5194)

### Mortality risk after confounder adjustment

We carried out multivariate Cox regression analyses to compare the mortality between different types of surgeries (Table 4). Laparoscopic surgery was associated with a significantly better survival than open surgery before and after confounder adjustment. Although robotic surgery was associated with a better survival than open surgery, the confidence interval was wide, and the survival benefit attenuated after confounder adjustment.

**Table 4:** Risk of mortality associated with the different surgeries before and after confounder adjustment.

	Laparoscopic vs. Open (HR, 95% confidence interval)	Robotic vs. Open (HR, 95% confidence interval)
Unadjusted analysis	0.781 (0.681, 0.893)	0.170 (0.055, 0.531)
Confounder adjusted analysis	0.801 (0.682, 0.950)	0.331 (0.105, 1.030)

### Overall mortality trends for the three different surgeries

**Figure 3:**
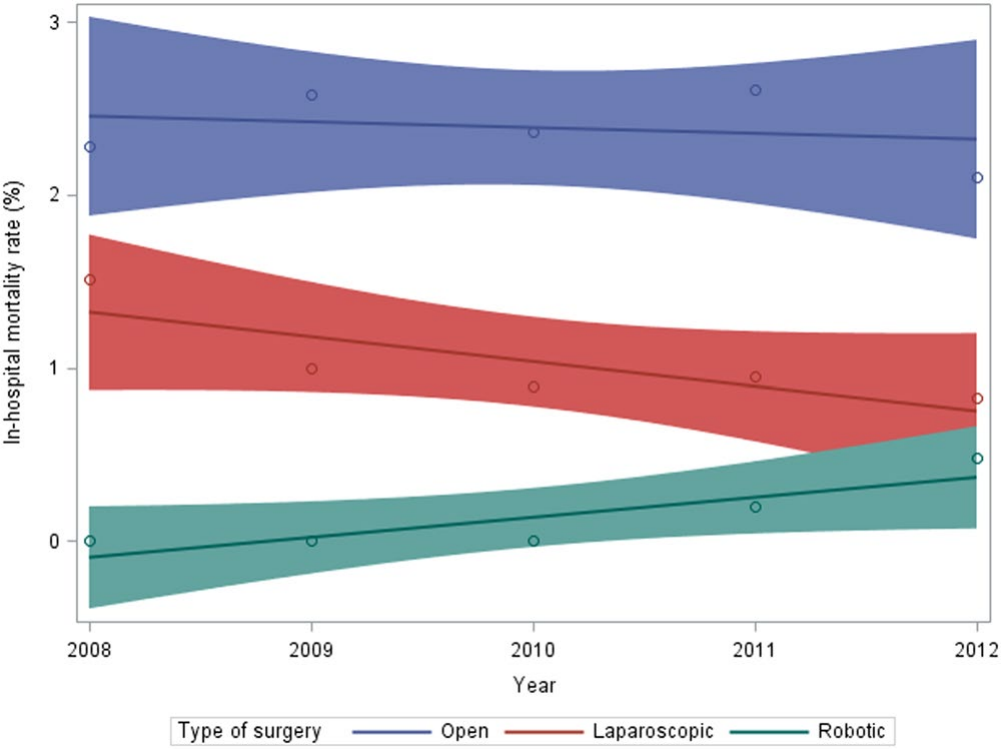
In-hospital mortality rate according to the type of surgeries, from 2008 to 2012. Circle represents the mortality rate in a given year, the straight line is a linear regression on all the data points, and the shaded area represents the 95% confidence limits for the linear regression.

Figure 3 compares the trend (Year 2008–2012) of in-hospital mortality rate associated with the different surgical treatment modalities. Overall mortality rate and the temporal trend in mortality vary substantially with the different surgical treatment modalities. Open surgeries were constantly found to have the highest overall in-hospital mortality rate (2.39%), followed by laparoscopic (0.95%) and finally robotic (0.26%). We observed a trend of decreasing in-hospital mortality rates for both open surgery and laparoscopic surgery. The in-hospital mortality rate for laparoscopic surgery is found to decrease by a faster annual rate as compared to open surgery (0.0036% per year vs 0.00138% per year). However, robotic surgery has a trend of increasing mortality rate, and the mortality rate increased from 0% in the year 2008 to 0.28% in the year 2012.

## Discussion

The number of admissions, regional nodal staging, and in-hospital mortality has changed substantially for colorectal cancer over the last decade. From 2004 to 2012, we found that there were a 13.5% decrease in the number of admissions, 46.4% decrease in the number of regional nodal cases, and 43.5% decrease in the number of in-hospital deaths. However, we did not observe a significant trend change in age, sex, and income of colorectal cancer patients during the study period. We observed that from 2008 to 2012, there was 35.4% decrease in the number of open surgeries, 3.5 fold increase in the number of laparoscopic surgeries (6536 to 29,105), and 41.3 fold increase in the number of colorectal robotic surgeries (74 to 3,130). A trend of decreasing in-hospital mortality rates for both open surgery and laparoscopic surgery was also observed. However, robotic surgery showed a trend of increasing mortality rate.

Our results on the decreasing annual trend of colorectal cancer admissions and overall in-hospital mortality concur with recent epidemiological studies. In fact, research conducted using the SEER Program registries found approximately a 27% decrease in the incidence rate and a 25% decrease in the mortality rate of colorectal cancer during our study period^1, 2^. Such results may reflect improvements in preoperative risk assessment, advances in intra and post-operative care, and an increase in adoption of FOBT and colonoscopy^29^. The guideline practice of screening people over 50 years of age with FOBT, followed, if positive, with a colonoscopy, has been associated with lower incidence rate and improvements in staging and mortality^3–5, 30–33^. In fact, we also observed a decrease in the number of node positive cases who carry the worse prognosis in terms of disease recurrence. Our observation is in accordance with the results observed in the SEER Program registries^2^. However, we must clarify that our numbers must not be directly compared to the results from the SEER Program registries. SEER Program registries collect personal data from 18 specific geographic areas across the United States, but NIS database collects deidentified hospital admission information from the entire 44 states. One reported issue with the SEER Program registries is that it is located in more urban and affluent areas than the non SEER Program registries area^34^. Although the NIS database samples in both urban and rural areas, there is no patient specific information to calculate incidence.

In addition, we found that the annual use of the three surgical has very different trends. Open surgery is still the most common surgical treatment throughout the study period, followed by laparoscopic and finally robotic surgeries. Although robotic surgeries only made up of 3.4% of the total number of colorectal surgeries performed in 2012, its use has grown exponentially by 40 folds. Our trend data on robotic surgery is consistent with older reports using the same NIS database^35, 36^. The observed trend increase has been explained by the pelvis being a rather narrow region to operate, and robotic surgery offers better vision than the other two surgical approaches. However, it is unclear how popular will robotic surgery becomes in the next decade, as there are a lot of cost issues and little demonstrated benefits. First, many hospitals show reluctance to acquire the robots, due to the high cost of buying ($1 to 2.5 million per unit) and maintaining these robots^37, 38^. Second, robotic surgery also results in a greater out-of-pocket expense for patients, which is a prohibitive factor for underinsured patient populations^38^. Third, several published studies did not find a clear outcome and cost benefit of robotic colorectal surgery over laparoscopic colorectal surgery^39, 40^.

Nevertheless, a potential benefit of robotic surgery over laparoscopic surgery is a decrease in the risk of in-hospital mortality. However, it is possible that the lower risk of robotic surgery is due to confounding by surgeons’ preference of indication, where they are unwilling to perform relatively high-risk colorectal patients with this new technology during their learning curve period. In fact, we observed a trend of increasing mortality rate for robotic surgery during the study period. Thus, further studies with a larger sample size, and a longer follow-up are required to clarify whether the observed decrease in risk of in-hospital death is true.

Another important observation is that the annual number of open and laparoscopic surgeries showed an opposing trend change. Several other researchers also reported a similar observation using the same NIS database, or other databases such as the Premier Inc, or the National Surgical Quality Improvement Program^14, 17, 41, 42^. However, we have a novel finding in the year 2012, which is the confidence limits for the weighted number of laparoscopic colorectal surgeries start to cross with the confidence limits of the weighted number of open colorectal surgeries. Given that several reports, including ours, found that laparoscopic colorectal surgeries have better short-term outcomes and lower cost than open surgeries, it is likely that laparoscopic surgeries may soon become the most commonly conducted type of colorectal surgery^18–20, 40^.

Results of our study should be interpreted in light of both strength and weakness. Our study’s strengths include a large nation-wide sample that closely represents the average colorectal patient. As far as we were aware of, we have identified the largest number of colorectal inpatients for subsequent surgical outcome comparison. The NIS database also includes virtually all hospital-based visits and is therefore not affected by selection bias stemming from selective inclusion of specific hospitals, health insurance systems, or age groups. By using a dataset of nationally representative hospitals, we were able to characterize the general adoption of MIS techniques for colorectal surgeries across a broader cohort of institutions, analyze the patterns of hospital care and costs, and result in several important findings.

There are also several inherent limitations in our study design. First, the NIS database lacks several patient and physician information that are required for investigating the actual causes for the changing trends in surgery. Like most electronic databases, NIS lacks detailed information on laboratory, pathology, imaging, and patient severity. These missing confounders might affect outcome comparison, as surgeons might select different procedures for patient with different severity, and different stage of disease. In fact, we observed that open surgery was selected against the other surgeries for regional or distant cancer, and emergency surgery. Second, NIS database lacks information on surgeon training and years of experiences, which may influence the choice of surgical procedure and the complication rates. Thus, we cannot rule out the possibility that surgeons who do not have a lot of experiences with minimally invasive surgeries selecting on the less severe patients. Finally, the NIS database is compiled from discharge data and does not have any outpatient follow-up data. Thus, the standard 30-day mortality or long-term length of survival after surgery cannot be captured in this database.

In conclusion, we found an extensive decrease in the number of colorectal cancer admissions, and its associated mortality over the last decade. We also observed a trend decrease in the number of open surgeries, and a corresponding trend increase in laparoscopic and robotic surgeries. Patient selection occurs for the different type of surgeries, and more population-based studies are required to clarify the cost-effectiveness associated with the different types of surgery.

## References

[1] Data, S. R. *SEER Stat Fact Sheets: Colon and Rectum Cancer*, http://seer.cancer.gov/statfacts/html/colorect.html (2010–2012).

[2] Siegel R, DeSantis C, Jemal A (2014). Colorectal cancer statistics, 2014. CA: a cancer journal for clinicians.

[3] Siegel RL, Ward EM, Jemal A (2012). Trends in colorectal cancer incidence rates in the United States by tumor location and stage, 1992-2008. Cancer Epidemiol Biomarkers Prev.

[4] National Center for Health, S. Health, United States, 2012: With special feature on emergency care (2013). 23885363

[5] Edwards BK (2010). Annual report to the nation on the status of cancer, 1975-2006, featuring colorectal cancer trends and impact of interventions (risk factors, screening, and treatment) to reduce future rates. Cancer.

[6] Phillips KA (2007). Trends in colonoscopy for colorectal cancer screening. Medical care.

[7] Meissner HI, Breen N, Klabunde CN, Vernon SW (2006). Patterns of colorectal cancer screening uptake among men and women in the United States. Cancer Epidemiology Biomarkers & Prevention.

[8] Bostick RM (1994). Sugar, meat, and fat intake, and non-dietary risk factors for colon cancer incidence in Iowa women (United States). Cancer Causes & Control.

[9] Sloane, D. In *Cancer Epidemiology* 65–83 (Springer, 2009).

[10] André T (2009). Improved overall survival with oxaliplatin, fluorouracil, and leucovorin as adjuvant treatment in stage II or III colon cancer in the MOSAIC trial. Journal of Clinical Oncology.

[11] Kopetz S (2009). Improved survival in metastatic colorectal cancer is associated with adoption of hepatic resection and improved chemotherapy. Journal of Clinical Oncology.

[12] Cone MM (2011). Dramatic decreases in mortality from laparoscopic colon resections based on data from the Nationwide Inpatient Sample. Arch Surg.

[13] Alnasser M (2014). National disparities in laparoscopic colorectal procedures for colon cancer. Surg Endosc.

[14] Kang CY (2012). Laparoscopic colorectal surgery: a better look into the latest trends. Archives of surgery.

[15] Yeo H (2015). Incidence of Minimally Invasive Colorectal Cancer Surgery at National Comprehensive Cancer Network Centers. Journal of the National Cancer Institute.

[16] Simorov A (2012). Laparoscopic colon resection trends in utilization and rate of conversion to open procedure: a national database review of academic medical centers. Annals of surgery.

[17] Bardakcioglu O, Khan A, Aldridge C, Chen J (2013). Growth of laparoscopic colectomy in the United States: analysis of regional and socioeconomic factors over time. Annals of surgery.

[18] Aziz O (2006). Laparoscopic versus open surgery for rectal cancer: a meta-analysis. Annals of surgical oncology.

[19] Ohtani H (2012). A meta-analysis of the short-and long-term results of randomized controlled trials that compared laparoscopy-assisted and open colectomy for colon cancer. Journal of Cancer.

[20] Abraham NS, Young JM, Solomon MJ (2004). Meta-analysis of short-term outcomes after laparoscopic resection for colorectal cancer. British journal of surgery.

[21] Cadiere GB (2001). Feasibility of robotic laparoscopic surgery: 146 cases. World J Surg.

[22] Delaney CP, Lynch AC, Senagore AJ, Fazio VW (2003). Comparison of robotically performed and traditional laparoscopic colorectal surgery. Diseases of the colon & rectum.

[23] Taggarshe D, Attuwaybi BO, Butler BN (2014). Robotic Surgery in Colorectal Cancer. Austin J Cancer Clin Res.

[24] Healthcare, C. & Utilization, P. *Overview of the Nationwide Inpatient Sample* (*NIS*) (Healthcare Cost and Utilization Project, Agency for Health Care Policy and Research, 2007).

[25] HCUP. *Trend Weights for HCUP NIS Data*, http://www.hcup-us.ahrq.gov/db/nation/nis/trendwghts.jsp.

[26] Steele SR, Brown TA, Rush RM, Martin MJ (2008). Laparoscopic vs open colectomy for colon cancer: results from a large nationwide population-based analysis. J Gastrointest Surg.

[27] Thompson NR (2015). A new Elixhauser-based comorbidity summary measure to predict in-hospital mortality. Med Care.

[28] Lin DY (2000). On fitting Cox’s proportional hazards models to survey data. Biometrika.

[29] van Vugt JL, Reisinger KW, Derikx JP, Boerma D, Stoot JH (2014). Improving the outcomes in oncological colorectal surgery. World J Gastroenterol.

[30] Kronborg O, Fenger C, Olsen J, Jørgensen OD, Søndergaard O (1996). Randomised study of screening for colorectal cancer with faecal-occult-blood test. The Lancet.

[31] Hardcastle JD (1996). Randomised controlled trial of faecal-occult-blood screening for colorectal cancer. The Lancet.

[32] Winawer S (2003). Colorectal cancer screening and surveillance: clinical guidelines and rationale-Update based on new evidence. Gastroenterology.

[33] Moiel D, Thompson J (2011). Early detection of colon cancer-the kaiser permanente northwest 30-year history: how do we measure success? Is it the test, the number of tests, the stage, or the percentage of screen-detected patients. Perm J.

[34] Nattinger AB, McAuliffe TL, Schapira MM (1997). Generalizability of the surveillance, epidemiology, and end results registry population: factors relevant to epidemiologic and health care research. J Clin Epidemiol.

[35] Halabi WJ (2013). Robotic-assisted colorectal surgery in the United States: a nationwide analysis of trends and outcomes. World J Surg.

[36] Moghadamyeghaneh Z, Phelan M, Smith BR, Stamos MJ (2015). Outcomes of Open, Laparoscopic, and Robotic Abdominoperineal Resections in Patients With Rectal Cancer. Dis Colon Rectum.

[37] Trinh, B. B., Hauch, A. T., Buell, J. F. & Kandil, E. Robot-assisted versus standard laparoscopic colorectal surgery. *JSLS* **18**, 10.4293/JSLS.2014.00154 (2014). 10.4293/JSLS.2014.00154 PMC4254475 25489211

[38] Salman M (2013). Use, cost, complications, and mortality of robotic versus nonrobotic general surgery procedures based on a nationwide database. The American surgeon.

[39] Keller DS, Senagore AJ, Lawrence JK, Champagne BJ, Delaney CP (2014). Comparative effectiveness of laparoscopic versus robot-assisted colorectal resection. Surg Endosc.

[40] Juo Y-Y (2014). Is minimally invasive colon resection better than traditional approaches?: First comprehensive national examination with propensity score matching. JAMA surgery.

[41] Delaney CP, Chang E, Senagore AJ, Broder M (2008). Clinical outcomes and resource utilization associated with laparoscopic and open colectomy using a large national database. Annals of surgery.

[42] Ozhathil DK (2011). Colectomy performance improvement within NSQIP 2005–2008. Journal of Surgical Research.

